# Case report: Immune checkpoint inhibitor-induced paraneoplastic neurological syndrome in two patients: a case series

**DOI:** 10.3389/fonc.2024.1404829

**Published:** 2024-10-28

**Authors:** Guang-Qing Shi, Heng-Ning Lian, Huan Wang, Jie-Qiang Xia, Li-Jie Ma, Jing Zhou

**Affiliations:** ^1^ Department of Respiratory and Critical Care Medicine, The General Hospital of Western Theater Command, Chengdu, China; ^2^ Department of Neurology, The First People’s Hospital of Shuangliu District, Chengdu, Sichuan, China

**Keywords:** anti-γ-aminobutyric acid B receptor antibody, anti-Hu antibody, immune checkpoint inhibitors, durvalumab, adebrelimab, paraneoplastic neurological syndrome

## Abstract

Immune checkpoint inhibitors (ICIs) combined with chemotherapy have improved overall survival in patients with small-cell lung cancer, but have also led to an increase in adverse effects. The incidence of ICI-induced paraneoplastic neurological syndrome (PNS) is relatively low when the primary lung lesion is well controlled. However, it is associated with high mortality and disability rates. In this report, we present two cases of extensive-stage small-cell lung cancer with neurological symptoms and positive paraneoplastic antibodies in the serum and cerebrospinal fluid (CSF) following ICI therapy. Although the symptoms improved after treatment with systemic high-dose immunoglobulin and glucocorticoids, one patient, unfortunately, succumbed to tumor progression four months later, whereas the other patient experienced persistent difficulty in standing and walking despite improved muscle strength. In cases where neurological symptoms that cannot be explained by tumor metastases arise during ICI treatment, paraneoplastic syndromes should be considered and testing for antineuronal antibodies is crucial, as early detection and intervention can help mitigate their impact. Further research is needed to develop better predictive strategies and treatment protocols for these adverse reactions.

## Introduction

Extensive-stage small-cell lung cancer (ES-SCLC) is highly malignant and has a short survival period, posing challenges to treatment. Immune checkpoint inhibitors (ICI) enhance the anti-tumor responses of immune cells by blocking immune checkpoint molecules such as programmed cell death 1 (PD1), programmed death ligand 1 (PD-L1), and cytotoxic T lymphocyte-associated protein 4 (CTLA4) (1). PD-L1 inhibitors such as atezolizumab, durvalumab, and adebrelimab, when combined with platinum and etoposide as first-line treatment, have shown promising results in improving the overall survival of patients with ES-SCLC (2–4). However, these medications can lead to adverse effects, including neurological-related events affecting 1-12% of patients (5, 6).

Paraneoplastic Neurological Syndromes (PNS) constitute a group of symptomatic, non-metastatic neurological diseases linked to systemic tumors, characterized by a pathophysiological mechanism involving a specific immune response targeted at antigens/epitopes common to both neoplastic and normal cells within the nervous system (7). ICI mainly enhances immune system resistance, activates T cells to fight cancer cells, and promotes their death through different immune-mediated pathways. This process inevitably produces immune-mediated adverse events. Immune-related adverse events mainly include diseases of other organs and systems, including PNS (8, 9). Among cancer patients receiving immune checkpoint inhibitor (ICIs) therapy, the overall incidence rate of PNS is less than 1.0% (10); nonetheless, within all immune-related neurological adverse events (ir-nAEs), PNS accounts for 15-20% (11). In some cases, n-irAEs may satisfy the clinical diagnostic criteria for PNS associated with “high-risk” antibodies (1). PNS has a higher mortality and disability rate (12). Therefore, when neurological symptoms manifest after ICI treatment initiation, it is important to consider PNS induced by ICIs during screening. Timely detection of neuronal antibodies and treatment with glucocorticoids, plasma exchange, intravenous immunoglobulins, and cyclophosphamide should be implemented (13). This study presents two cases of patients with ES-SCLC who initially did not exhibit neurological symptoms. Following treatment with a PD-L1 inhibitor, both patients developed PNS. Tragically, one patient succumbed to disease progression after 4 months, whereas the other experienced debilitating neuropathy.

### Case 1

**Figure 1:**
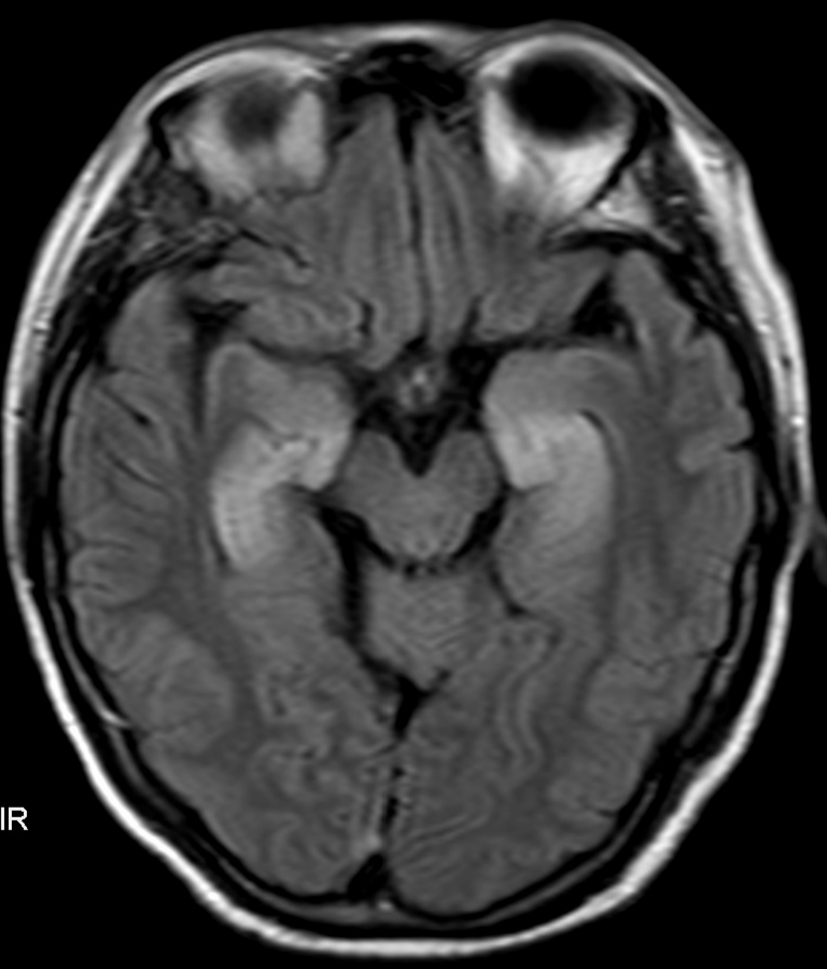
Fluid attenuation inversion recovery (FLAIR) magnetic resonance imaging of the head revealed high signal areas in the bilateral medial temporal lobe and hippocampus.

A 58-year-old female patient was admitted on September 7, 2021, because of shortness of breath, dizziness for 1 week, nausea, and vomiting for 4 days. The diagnosis was SCLC in the right lung with metastases to the mediastinum, right hilum, neck, axillary lymph nodes, and right pleura classified as T4bN3M1a (extensive stage). Starting on September 15, 2021, 120 mg etoposide on Days 1-3 plus 90 mg cisplatin on Day 1 via intravenous drip (every 3 weeks) was administered for six cycles. On January 19, 2022, an additional 1000 mg of D1 Q4W treatment with durvalumab (AstraZeneca UK Limited) was initiated. During this period, regular follow-up evaluations revealed partial remission (PR). After 19 cycles of durvalumab (July 1, 2023), the patient experienced seizures characterized by loss of consciousness, convulsions, foaming at the mouth, and clenched teeth lasting for approximately 1 min. Subsequently, progressive cognitive decline, increased sleep, decreased speech, and olfactory hallucinations occurred. After admission, the electrolyte test results showed Na+ at 114.2 mmol/L and Cl- at 82.2 mmol/L. Electroencephalography (EEG) revealed mild-to-moderate abnormalities characterized by an increase in slow waves. Fluid attenuation inversion recovery (FLAIR) magnetic resonance imaging of the head revealed high signal areas in the bilateral medial temporal lobe and hippocampus (**Figure 1**). A Mini-Mental State Examination score of 14/30 indicated moderate cognitive impairment. Lumbar puncture revealed a normal cerebrospinal fluid (CSF) cell count and protein and sugar levels. No malignant cells were found on the CSF cytological examination. Considering the possibility of encephalitis and paraneoplastic syndrome, CSF and serum samples were sent to the testing center for analysis. The results showed that oligoclonal bands were observed in serum and CSF. Positive anti-GABA_B_R antibodies were detected in the serum and CSF samples. Paraneoplastic syndrome antibodies (anti-Hu, anti-Yo, anti-Ri, anti-CV2, anti-amphiphysin, anti-Ma1, anti-Ma2, anti-SOX1, anti-DNER, anti-Zic4, anti-GAD65, anti-PKC, anti-recoverin, and anti-titin) were negative in the serum and CSF, whereas other autoimmune encephalitis antibodies (anti-NMDA, anti-LGI1, anti-CASPR2, anti-AMPAR1, and anti-AMPAR2) were negative in the serum and were not detected because of insufficient CSF samples. On the basis of these results, the patient was diagnosed with durvalumab-induced anti-GABA_B_R antibody-associated encephalitis. Therefore, durvalumab was discontinued and the patient began receiving 22.5 g of immunoglobulin (0.4 g/kg) daily and 1000 mg of methylprednisolone sodium succinate daily for 5 days. Additionally, 0.25 g of levetiracetam was administered twice daily to prevent seizures and correct electrolyte imbalance. After medication, the patient did not experience seizures, but cognitive impairment gradually increased. The patient finally died on November 2023 owing to pancreatic metastasis from SCLC.

### Case 2

A 70-year-old male patient was admitted to the hospital on December 15, 2023, because of a cough that persisted for 10 days. The diagnosis was SCLC in the right lung with mediastinum, right hilar lymph node, and intracranial metastasis classified as T4N3M1a (extensive stage). Starting on December 21, 2023, a combination of immunotherapy and chemotherapy was administered: adebrelimab injection (Aireli, Hengrui Pharmaceutical Company) at 1200 mg on Day 1, 120 mg etoposide on Days 1-3, and 200 mg carboplatin on Day 1 (every three weeks) for two cycles. On April 23, 2024, chest CT efficacy evaluation showed a partial response. Following the completion of the second treatment cycle, the patient experienced weakness and numbness in the upper limbs, progressively worsening symptoms, an inability to stand or walk, and difficulty in making a fist with both hands. Physical examination revealed bilateral upper limb muscle strength is three out of five and lower limb muscle strength is two out of five. Sensory examinations were normal, including pain, temperature, vibration, and position sense. He did not have a Babinski sign or a sign of an exaggerated deep tendon reflex. Cranial and cervical MRI scans were negative for space-occupying lesions. A nerve conduction study showed lower amplitude in the right median nerve, bilateral tibia, and bilateral common peroneal nerve CMAP and reduced conduction velocity in the right common peroneal nerve, whereas SNAPs were normal. The F wave showed a decrease in the outgoing wave rate of the bilateral median nerve and bilateral tibial nerves. Lumbar puncture revealed normal pressure with a total CSF protein of 0.97 g/L(0.01-0.45 g/L) and nucleated cell count of 1.0 * 10^6^/L(0-8.0*10^6/L). CSF cytology, culture, and smear were all negative. No tumor cells were found upon examination of the CSF shed cells. Complete sets of serum autoimmune and ANCA antibodies were negative. The CSF and serum testing center detected that IgG oligoclonal bands in the CSF were similar in type IV CSF and serum. IgG 24-h intrathecal synthesis rate at 10.04 mg/24 h(< 7mg/24 h). Ganglioside antibody detection: IgM was positive for anti-GM1, anti-GM2, and anti-GD2 antibodies. The results of paraneoplastic syndrome antibodies in CSF and serum were: anti-Hu antibodies were positive, whereas anti-Ri, anti-CV2, anti-amphiphysin, anti-Ma1, anti-Ma2, anti-SOX1, anti-DNER, anti-Zic4, anti-titin, and-recovery, anti-PKC, anti-GAD65, and anti-Yo antibodies were negative. Based on these results, the diagnosis was anti-Hu positivity associated with adebrelimab-induced paraneoplastic neuropathy. Therefore, adebrelimab was discontinued and methylprednisolone sodium succinate at 1000 mg/day plus immunoglobulin (0.4 g/kg/d) 20 g/day was administered for 5 days. After treatment, the patient’s bilateral upper limb muscle strength was four out of five, lower limb muscle strength was three out of five, and symptoms improved; however, the patient was still unable to stand and walk.

## Discussion

ICI-associated adverse events and toxicities are commonly referred to as immune-related adverse events (ir-AEs). The most frequent ir-AEs include hypothyroidism, pneumonia, rash, diarrhea, colitis, and hepatitis (14, 15). Although immune-related neurological adverse events (ir-nAEs) are rare, they typically occur 3 to 4 months post-ICI treatment or within 12 months after the last infusion (8). These events are often severe, leading to a significant decline in the patients’ quality of life and, in some cases, short-term mortality (16). Therefore, early recognition of this condition is of paramount importance. Ir-nAEs can present in various forms such as irMeningitis, irEncephalitis, irDemyelinating disease, irVasculitis, irNeuropathy, irNeuromuscular junction disorders and irMyopathy (8, 17). Although neurological adverse events have been observed in phase III trials of atezolizumab and durvalumab in ES-SCLC treatment, no such events have been reported with adebrelimab (2–4). The two cases reported here experienced severe ir-nAEs after using ICIs; Case 2 was the first reported ir-nAEs after using adebrelimab, and Case 1 developed borderline encephalitis and tested positive for anti-GABA_B_R antibodies 17 months after maintenance treatment with ICI. Case 2 developed progressive bilateral symmetrical limb weakness, separation of the CSF protein cells, and peripheral nerve axonal degeneration with demyelinating changes, primarily axonal damage. Anti-GM1, anti-GM2, and anti-GD2 IgM antibodies were positive, anti-Hu antibodies were positive after two cycles of ICI treatment, and the final diagnosis was PNS. There are some limitations to the present case report. Autoimmune encephalitis antibodies (anti-NMDA, anti-LGI1, anti-CASPR2, anti-AMPAR1, and anti-AMPAR2) were not detected because of insufficient CSF samples

PNS is a group of disorders linked to cancer that can affect different parts of the central or peripheral nervous system. These conditions do not stem from cancer spreading within the nervous system or local effects but rather from an immune response triggered by cancer that targets neuronal proteins (18). Antibodies related to paraneoplastic syndromes fall into two categories: those against intracellular neuronal proteins such as anti-Hu/ANNA1,anti-Ri/ANNA2,and anti-Yo, are linked to specific PNS and mediated by T lymphocyte cytotoxic effects; and those against synaptic or cell membrane proteins such as anti-NMDA-R, anti-AMPA-R, anti-GABAB-R, which directly cause disease (19). For instance, antibodies such as anti-Hu/ANNA1,anti-SOX1,anti-Amphiphysin, and anti-amphiphysin are commonly associated with SCLC (1, 20), whereas anti-AMPA-R and anti-Ma2 antibodies are primarily linked to non-small cell lung cancer (21, 22). Furthermore, a positive antibody in the PNS (e.g., anti-Hu, anti-Yo, or anti-Ma2) indicates a poor response to treatment, whereas a positive anti-NMDA antibody suggests a better treatment response (1, 23, 24). Case 2 tested positive for anti-Hu antibodies, resulting in poor treatment response and an inability to stand and walk. It is noteworthy that both cases in this report employed corticosteroids and intravenous immunoglobulin therapies. Should these treatments prove insufficiently efficacious, research suggests exploring the use of monoclonal antibodies such as Natalizumab, Rituximab, and others as potential alternatives. This direction constitutes a primary focus of future research in the treatment of PNS (10).

ICIs can trigger or exacerbate antibody-or T cell-mediated PNS (23). PNS is observed in patients undergoing ICI treatment, suggesting that ICIs may catalyze PNS, given the absence of neurological symptoms before immunotherapy. Although spontaneous PNS typically arises in untreated patients in the early stages of cancer diagnosis, ICI-induced PNS is more prevalent in patients with advanced cancer diagnoses and improved primary tumor control after treatment (17). Patients with myasthenia gravis, myositis, or paraneoplastic encephalitis are at higher risk of recurrence of their underlying neurological diseases after ICI initiation, leading to significant incidence rates and mortality (25). High-risk presentations of PNS include Limbic encephalitis,Paraneoplastic cerebellar degeneration, Sensory neuronopathy, Enteric neuropathy, Paraneoplastic encephalomyelitis, Opsoclonus myoclonus syndrome, and Lambert-Eaton syndrome (1). Given that many PNS symptoms overlap with tumor-related effects, intracranial metastasis, chemotherapy side effects, and non-specific conditions, such as muscle weakness and fatigue, identifying the association with ICIs can be challenging, leading to delayed diagnosis, treatment, and potential oversight. Previous studies have indicated that following treatment with ICIs, anti-GABAB-R antibody-associated encephalitis and anti-Hu antibody syndrome exhibit diverse clinical manifestations (**Table 1**) (26–29). Case1 developed limbic encephalitis after 17 months of continuous ICI therapy, a timeframe divergent from the previously documented 3-4 months in literature, potentially attributable to variations in follow-up duration or sample sizes in studies. In contrast, Case 2 exhibited concurrent impairment of sensory and motor nervous systems, resembling the less common Guillain-Barré Syndrome (30), as opposed to the more prevalent subacute sensory neuronopathy seen in prior anti-Hu antibody syndrome cases. The role of as yet undisclosed positive antibodies in this discrepancy hints at a need in future research to broaden antibody testing scopes and amass more instances to identify frequently occurring antibody types, thereby furnishing a more cost-effective diagnostic panel for related adverse reactions. From a pharmaceutical application standpoint, PNS cases triggered by durvalumab largely manifest as limbic encephalitis. Consequently, throughout the course of ICI-based cancer therapies, vigilant monitoring for any neural adverse effects is paramount, regardless of treatment duration, symptom specificity, or chosen medication. Enhancing comprehension of such cases necessitates the accumulation and meticulous examination of an expanded case database.

**Table 1:** Paraneoplastic neurological syndromes with anti-GABA_B_R, anti-Hu antibodies after immune checkpoint inhibitor treatment.

Sex, age (years)	Tumor	ICI (number of cycles)	Time from first dose of ICI to irnAE (weeks)	Time from tumor diagnosis to ir-nAE (months)	ir-nAEs	Antibody Type	Treatment	References
F,56	SCLC	Durvalumab (19)	93	22	LE	anti-GABA_B_R	Methylprednisolone, IVIG	Present series
M,61	SCLC	Durvalumab (2)	6	2	LE	anti-GABA_B_R	Methylprednisolone, IVIG	Li et al. (26)
M,72	SCLC	Durvalumab (8)	41	9	LE	anti-GABA_B_R_1_	Methylprednisolone, plasmapheresis	Moss et al. (27)
F,66	SCLC	Durvalumab (3)	10	2	AE	anti-GABA_B_R	Methylprednisolone	Shechtman et al. (28)
M,70	SCLC	Adebrelimab (2)	6	2	PN	anti-Hu	Methylprednisolone, IVIG	Present series
M, 46	SCLC	Pembrolizumab (4)	12	5	SSN	anti-Hu	Methylprednisolone, IVIG	Mongay-Ochoa et al. (29)
M, 71	SCLC	Atezolizumab (3)	8	4	CS	anti-Hu	IVIG	Mongay-Ochoa et al. (29)

ICI immune checkpoint inhibitor, IVIG intravenous immunoglobulin, ir-nAE neurological immune-related, F female, M male, SCLC small cell lung cancer, NSCLC non-small cell lung cancer, LE limbic encephalitis, AE autoimmune encephalitis with extralimbic involvement, PN: Peripheral neuropathy, SSN sensory neuronopathy, CS cerebellar syndrome.

Patients with PNS following ICI treatment should be managed consistently according to ir-nAEs guidelines; any PNS can lead to significant neurological impairment and is therefore classified as a grade 3-4 irAE, necessitating the immediate discontinuation of ICI therapy (31). Resuming ICI therapy in the context of persistently improving neurological symptoms post-treatment has emerged as a contentious topic in the medical realm. At the heart of clinical decision-making lies the delicate balance between risks and benefits for each patient, with pivotal considerations encompassing: the patient’s projected survival time, the magnitude of neuroimmune-related adverse events (ir-nAEs), the history of response to prior ICI treatment, and the availability of alternative oncological therapeutic options. To minimize the risk of recurrent ir-nAEs, some clinicians favor a concurrent approach of using corticosteroids or exploring alternative immunosuppressive agents when reinstating ICI therapy (32). Dalakas.M.C believes that it is possible to try to change the type of ICIs, because using a different ICI may be an evolving option because ir-AE associated with one class of ICIs (e.g. anti-CTLA-4) may not necessarily recur with another class (e.g. anti-PD-1) (33). Additional long-term data, especially from prospective studies, are imperative to thoroughly address the concerns posed by both viewpoints. In the absence of clear directive principles, the therapeutic strategies crafted through collaboration between seasoned oncologists and neurology experts become invaluable to patient care. Unfortunately, within the confines of this study, neither of the two case patients resumed treatment with immune checkpoint inhibitors (ICIs); most grievously, patient one succumbed to disease progression, an outcome deeply lamented.

In clinical practice, it is common to monitor autoimmune antibodies in the patient’s blood while using ICIs to prevent autoimmune diseases such as lupus erythematosus and rheumatoid arthritis. However, the detection of central nervous system antibodies is not routinely performed owing to costs and other factors. With the increasing use of ICIs in cancer treatment, there is a need to discuss whether monitoring these antibodies is necessary for the early identification of populations at high risk for PNS. The study encounters a notable limitation as neither of the reported cases included pre-ICI therapy assessment for neural antibodies. Refining the sentence: Determining whether pre-existing positive PNS antibodies escalate the risk of PNS-like neurotoxicities subsequent to ICI therapy, alongside uncovering additional predictive biomarkers for PNS incidence and outcome (17), extending beyond neural antibody assessments – including markers like Neurofilament light chain (NfL), Interleukin-6 (IL-6), etc (34). – constitutes a paramount focus of ongoing research efforts. In previous drug trials, central nervous system adverse reactions from different mechanisms were often grouped under the same disease category, lacking precision in drug selection, owing to insufficient recognition and reporting of autoantibody-induced categories. Addressing this issue should be considered in future experimental drug design.

ICI-induced PNS is characterized by rapid and widespread neuronal loss driven by T cells. Early identification and treatment are essential to prevent irreversible neurological disability (17). The prognosis of PNS may be poor and requires aggressive treatment (5). Consequently, it is imperative to consider PNS in all cancer patients exhibiting neurological symptoms during ICI therapy, with immediate initiation of neuroantibody testing to expedite timely intervention.

## Data Availability

The raw data supporting the conclusions of this article will be made available by the authors, without undue reservation.
